# A peculiar case of psychosis: anti-NMDAr encephalitis

**DOI:** 10.1186/s12245-021-00389-y

**Published:** 2021-11-04

**Authors:** Lamia Kouba, Dalia Alhosain

**Affiliations:** 1 Department of Dermatology, Damascus University Hospitals, Damascus, Syria; 2 Department of Internal Medicine, Damascus University Hospitals, Damascus, Syria

**Keywords:** Anti-NMDA receptor encephalitis, Herpes simplex virus, Psychosis, Pregnancy, Case report

## Abstract

**Background:**

Psychosis in pregnancy is rare and could be life-threatening. It requires prompt evaluation and proper management accordingly. Anti-N-methyl-d-aspartate receptor (anti-NMDAr) encephalitis following herpes simplex virus (HSV) infection is a rare cause of psychosis during pregnancy.

**Case presentation:**

A 20-year-old woman at 18 weeks gestation presented with agitation and a 3-day history of hallucinations. She had a prior hospitalization for HSV encephalitis 6 weeks before. Her laboratory workup was unremarkable except for positive anti-NMDAr antibodies in the CSF. The patient was treated with high-dose corticosteroids and plasmapheresis, and she was discharged 2 weeks later fully recovered.

**Conclusions:**

Anti-NMDAr encephalitis can be the culprit behind a new-onset of psychosis in pregnancy. Early diagnosis and treatment are crucial.

## Background

Anti-N-methyl-d-aspartate receptor (anti-NMDAr) encephalitis is the most common autoimmune encephalitis. It was first reported in 2005 and has been thoroughly studied since then [1].

It is postulated that autoantibodies target the Nu1 subunit of the NMDA receptor, decreasing its activity, and leading to symptoms of anti-NMDAr encephalitis. Anti-NMDAr encephalitis presentation often comprises prodromal viral-like symptoms that progress into psychiatric symptoms, confusion, memory disturbances, seizures, and movement disorders.

The two known triggers of anti-NMDAr encephalitis are tumors and infectious agents, particularly the herpes simplex virus (HSV).

Anti-NMDAr encephalitis is rare in pregnancy, and its psychiatric features resemble many primary mental disorders that could present with acute psychosis.

We report the case of a young pregnant woman presenting with an acute psychotic episode 3 weeks after recovering from HSV encephalitis. Her acute symptoms were attributed to anti-NMDAr encephalitis following HSV-1 infection. We believe that this report sheds some light on this recently described disease and encourages clinicians to consider this diagnosis when evaluating acute episodes of psychosis in pregnant patients.

## Case presentation

A 20-year-old female gravida 2 para 1 at 22 weeks gestation presented to our emergency room with agitation. She experienced 5 days of visual hallucinations according to her family. They denied any fever, headache, or vomiting. They also denied any recent history of head trauma. The patient does not use tobacco, alcohol, or illicit drugs. She had a normal prenatal course during the first trimester.

Six weeks earlier, she presented to our institution with a 3-day history of severe headache, fever, and vomiting accompanied by behavioral changes for 2 days. She was diagnosed with HSV-1 encephalitis based on positive HSV1 PCR in the cerebrospinal fluid and characteristic brain MRI findings. She was treated with acyclovir for 3 weeks and was symptom-free at discharge.

Based on the patient’s history and current presentation, a CSF sample was obtained to rule out HSV encephalitis recurrence. Cerebrospinal fluid was within normal limits, and HSV1 PCR was negative. Laboratory panels including liver, renal, and thyroid function tests, electrolyte concentrations, full blood count, and coagulation markers were all within normal limits. Vitamin B_12_ and folic acid levels were also normal.

Subsequently, herpes simplex virus-induced anti-N-methyl-d-aspartate (NMDA) receptor encephalitis was suspected. CSF anti-NMDA receptor antibodies (NMDAR Ab) were positive.

An abdominal ultrasound was performed to rule out ovarian teratoma, which is known to cause anti-NMDAr encephalitis. It showed a 22-week-old viable fetus, and no pelvic masses were seen.

Treatment with high-dose corticosteroid was started, and plasmapheresis was performed. The patient had remarkable improvement within 2 weeks of admission. She was discharged fully recovered.

## Discussion

When evaluating a patient with acute altered mental status or psychosis, the primary goal is to differentiate between encephalopathy and a psychiatric disorder such as schizophrenia or bipolar disorder [2].

It is very unlikely for a first psychotic episode of a psychiatric disorder to occur during pregnancy [3]. A new-onset of psychosis in a pregnant woman should stimulate a thorough workup for an underlying medical or pharmacologic etiology, particularly when it is accompanied by clear disorientation, visual or tactile hallucinations, fluctuations in the level of consciousness, or evident neurologic symptoms [2], whereas psychotic episodes of psychiatric disorders present with delusions, hallucinations, thought disorganization, and agitation [4].

There is limited data on the incidence of new-onset psychotic episodes during pregnancy. According to Paffenbarger et al., the overall rate of mental illness during pregnancy was 5 per 10,000 live births [3].

However, psychotic episodes of the underlying psychiatric disease may relapse or reactivate during pregnancy [2, 3].

Psychosis can be the presenting symptom of many medical illnesses and neurologic conditions, such as delirium, endocrine disorders (thyroid and adrenal dysfunction), hepatic and uremic encephalopathy, infectious disease, inflammatory and demyelinating disorders, metabolic disorders, neurological and neurodegenerative diseases, and vitamin deficiency.

Our patient had no history of endocrine, renal, or hepatic disease, and her liver and renal function tests were normal at the current presentation. She had no history of alcohol abuse, and she had no diagnosed psychiatric disease.

However, she had a history of HSV encephalitis 6 weeks earlier and was treated with acyclovir until complete resolution. Based upon her current symptoms and history, it was presumed that she was either undergoing a recurrence of the viral encephalitis or autoimmune encephalitis following the viral disease.

Her CSF sample analysis confirmed the latter, and HSV-induced anti-NMDAr encephalitis was diagnosed.

To our knowledge, this is the first reported case in Syria of anti-NMDAr encephalitis following HSV infection in a pregnant patient.

Anti-NMDAr encephalitis is rarely reported in pregnancy, and when it occurs, it is difficult to delineate it from other neuropsychiatric disorders.

Rare disorders as such can only be diagnosed when they are considered: therefore, we highlight the importance of taking a detailed patient history when evaluating psychosis in a pregnant woman.

The clinical presentation of anti-NMDAr encephalitis comprises a viral-like illness that later progresses to a constellation of symptoms including, but not limited to, psychiatric manifestations, seizures, decreased consciousness level, dyskinesias, language defect, and autonomic dysfunction.

Anti-NMDAr is associated with the presence of underlying tumors, namely ovarian teratomas containing nervous tissue [5]. In a cohort study conducted by Titulaer et al., it was found that 50% of anti-NMDAr female patients had ovarian teratomas [6]. However, in patients older than 45 years, anti-NMDAr encephalitis is rarely accompanied by tumors, and in those cases, carcinomas are more commonly detected leading to a less favorable outcome [7].

Anti-NMDAr encephalitis is also associated with preceding herpes simplex encephalitis. Studies have shown that 20–30% of patients with prior HSV encephalitis who present with a relapse of symptoms not related to HSV relapse do have anti-NMDAr antibodies detected in their CSF [8].

Treatment options for anti-NMDAr encephalitis include tumor resection and immunosuppressive therapy. Intravenous methylprednisolone, intravenous immunoglobulin G, and plasma exchange represent the first-line treatment options. If no clinical improvement is observed, rituximab and cyclophosphamide are suggested as the second-line therapies.

Anti-NMDAr IgG antibodies can cross the placenta and have been reported to be detectable in the serum of babies born to affected mothers during pregnancy. However, it is still unclear whether these autoantibodies have noxious effects on the fetal outcome.

We report the case of an acute psychotic episode during pregnancy due to a rare etiology.

## Data Availability

Not applicable.
